# Effectiveness of training in expressing positive emotions, reacting to change and greeting peers after childhood traumatic brain injury: a single-case experimental study

**DOI:** 10.3389/fpsyg.2023.1195765

**Published:** 2023-07-12

**Authors:** Sandra Rivas-García, Nuria Paúl, Andrés Catena, Alfonso Caracuel

**Affiliations:** ^1^Area of Developmental and Educational Psychology, Department of Psychology, University of Cadiz, Cádiz, Spain; ^2^Mind, Brain and Behaviour Research Centre (CIMCYC), University of Granada, Granada, Spain; ^3^Department of Experimental Psychology, Complutense University of Madrid, Madrid, Spain; ^4^Department of Experimental Psychology, University of Granada, Granada, Spain; ^5^Department of Developmental and Educational Psychology, University of Granada, Granada, Spain

**Keywords:** social cognition, traumatic brain injury, single-case experimental design, theory of mind, intervention studies, emotion expression

## Abstract

**Background:**

Social cognitive deficits are common after traumatic brain injury (TBI). The participant in this single-case experimental design (SCED) was 7 years old when he sustained a severe TBI. After 2 years in rehabilitation, he continues to show deficits in social cognition.

**Objective:**

To determine the effectiveness of three interventions, each aimed at improving a behavior altered by social cognition deficits. These behaviors were: (1) expression of positive emotions, (2) reacting to changes in plans, and (3) greeting classmates.

**Method:**

An A-B-A’ design was used for each behavior. In addition, each behavior was targeted with a rehabilitation program applied over 10 sessions.

**Results:**

For the first behavior, changes between phases B-A’ (NAP = 0.712) and A-A’ (NAP = 0.864) indicated improvements in the child’s ability to express positive emotions. In the second behavior, changes in the intensity of reactions between phases B and A’ (NAP = 0.815) and A vs. A’ (NAP = 0.834) indicated that the child adapted to changes in a plan and to unexpected situations in a more adaptive way. For the third behavior, changes in the number of greetings between phases A and B (NAP = 0.883) and A vs. A’ (NAP = 0.844) suggested that during the third phase of the study, the participant fully acquired the habit of greeting peers and increased his interactions with others.

**Conclusion:**

While the participant showed improvements in all three targeted behaviors, due to the complexity of the third behavior, it is recommended that in future research, the intervention targeting social interactions should be applied over a longer timeframe to ensure that improvements are more stable in the long term.

## 1. Introduction

Social cognition (SC) is a complex capacity made up of skills related to emotion processing, social knowledge, theory of mind, and empathy (McDonald and Genova, 2021). All of them play a decisive role in social integration (Frith, 2008). Emotion processing is the capacity to discern and express emotions, while social knowledge is the ability to decode and interpret behavior in specific social situations, such as understanding social norms, roles, and goals and how these can influence the behavior of others. Theory of mind (ToM) is the ability to understand other people’s minds, including their interests, beliefs, emotions, and intentions. Finally, empathy allows us to respond to the thoughts and feelings of others with an appropriate emotion (Dennis et al., 2012; Byom and Mutlu, 2013; Neumann et al., 2022). Childhood is a key time in the development of these skills that people need to interact and function successfully in society (Bulgarelli et al., 2022). However, traumatic brain injury (TBI) at childhood is a leading cause of disability (Dewan et al., 2016) that have detrimental effects on cognition, emotion, behavior (Genova et al., 2019) and social functioning (Bedell and Dumas, 2004). After TBI, social impairment is often a consequence of social cognition deficits (Feeney and Achilich, 2014). The review by Ju et al. (2021) provides evidence that disruption in ToM plays a key role in the difficulties in social functioning of children and adolescents with TBI. Deficits in ToM have been found related to others cognitive problems such as dysexecutive behavior, and both are significant predictors of less social participation (Westerhof-Evers et al., 2017).

In addition to deficits in ToM, after TBI the person often shows deficits in the other components of CS (Tousignant et al., 2018). Emotion processing is impaired because the person shows difficulty recognizing emotional expressions from different media, including audio and visual channels, and still versus moving screens (Mcdonald and Saunders, 2005). In relation to social knowledge, Mah et al. (2005) demonstrated that deficits in this component contribute to the aberrant social behavior observed after brain injury. Finally, in relation of the empathy, Hillis’ review shows that people with brain injury have impaired this component (Hillis, 2014) and it has widespread and deep implications for social function in children with TBI (Dennis et al., 2013).

SC deficits persist for at least 2 years after TBI in one in four children (Anderson et al., 2017). Sirois et al. (2019) argue that there are shortcomings in the detection of these deficits, and therefore SC tasks should be included in assessment protocols employed following childhood TBI. In this regard the instruments available are questionnaires for parents such as the Children’s Empathy Quotient (EQ-C) (Auyeung et al., 2009) and performance-based instruments, for example, the ToMas-child (Rivas-Garcia et al., 2020) which measures theory of mind in children aged three to 7 years of age. Howewer, researchers have highlighted the need to develop more instruments to assess social cognition during childhood (On et al., 2022).

Concerning SC intervention approaches, most programs have been designed for people with autism or schizophrenia (Cassel et al., 2016; Loubat and Astudillo-Zúñiga, 2019). Three widely used programs focus on emotion recognition (Bornhofen and Mcdonald, 2008a; Murphy et al., 2021), —the Micro-Expression Training Tool (Russell et al., 2006), the Emotion Management Training (Hodel et al., 2004), the Program Emotion Training (Silver et al., 2004) and the Training of Affect Recognition (Wölwer et al., 2005). However, for people with TBI, there are far fewer programmes aimed at improving CS that have been shown to be effective (Rodríguez-Rajo et al., 2022). In addition, most programs only address the processing of emotions (Radice-Neumann et al., 2009; McDonald et al., 2013; Williasom and Isaki, 2015). The Reading a Smile program (Bornhofen and McDonald, 2010) is one of them. It has been shown to improve the ability of adults to judge basic emotional stimuli when presented in video format and to make social inferences based on the speaker’s behavior (Bornhofen and Mcdonald, 2008b). However, developing child-specific rehabilitation programs remains a key challenge due to most of them are aimed at adolescents and adults (Cassel et al., 2016). Another challenge, according to the review by Vallat-Azouvi et al. (2019), is the need to design interventions whose effect generalizes and improves social skills in everyday life. For this purpose, the single-case experimental design (SCED) — widely used in research with TBI patients (Penn et al., 2005; Nahum et al., 2014)— is now being extended to address SC deficits shown by such individuals (Rietdijk et al., 2019; Vassallo and Douglas, 2021). In particular, the A-B-A’ design has shown validity in intervention studies for improving social cognition (Peyroux and Franck, 2016), increasing social skills and decreasing challenging behaviors (Jacobson and Truax, 1992; Binder et al., 2019).

In summary, there is a need for programs that target SC problems in children with TBI, that take into account the different components of CS that are deficient and with an ecological approach through training in everyday life behaviors.

This study aimed to determine the efficacy of three interventions applied to a child with TBI, each targeting a specific behavior altered by social cognition deficits: (1) expression of positive emotions, (2) reacting to changes in plans, and (3) greeting peers.

## 2. Method

### 2.1. Design

The present study was designed following SCED guidelines (Kratochwill et al., 2013; Tate et al., 2013), taking into account the Risk of Bias in Trials Scale N of 1 (RoBiNT) (Tate et al., 2015) and the Single-Case Reporting Guidelines for Behavioral Interventions (SCRIBE) (Tate et al., 2016). The checklist with each of the items of the RoBiNT scale is specified in Supplementary Table 1, and those of the SCRIBE scale are shown in Supplementary Table 2.

The child’s parents provided written informed consent. Subsequently, an A-B-A’ fall-back/withdrawal design was applied, and the target behaviors were agreed upon following a discussion with the child’s family. The participant was blinded to the study design. At the baseline phase, the child was unaware that his behaviors were being recorded, one of which was captured by a video camera. This arrangement was used to avoid any intentional behavior modifications. At the beginning of the intervention phase, the family explained to the child that he would participate in a study aimed at people with TBI. However, neither the characteristics of the study nor the fact that certain behaviors would be assessed outside the session was explained to the child. Finally, the child did not know that his behaviors were being recorded in the withdrawal phase (as with the previous phases).

The participant received three different interventions, one for each target behavior. Each phase included a minimum of nine data points. We randomized the order of the three targeted behaviors and the starting day of the intervention phase for each, taking into account that a minimum of nine measures were required at baseline. The three interventions were developed sequentially. The baseline for the next intervention was not started until the follow-up phase was completed after the withdrawal of each intervention. In this way, the possible influence of each intervention on the next target behavior was controlled by checking the stability of the baseline for each behavior.

The researchers who explained the objectives to the parents also conducted the rehabilitation programs. Another researcher directed data collection and analyses. Neither was blinded to the phase of the study.

### 2.2. Participant

#### 2.2.1. Selection criteria

The researchers of this study provided the inclusion/exclusion criteria to one public pediatric hospital so that the center could use this information to refer potential participants. The inclusion criteria for this study were (1) being a child between the age of 5–15 years, (2) having suffered brain damage, (2) having impaired social cognition, and (3) having previously attended therapy. The exclusion criteria for this study were (1) having language problems and (2) having attention problems.

#### 2.2.2. Participant characteristics

David (pseudonym) is a nine-year-old boy who lives with his parents and siblings. He is the middle child and has close daily contact with other family members, such as his grandparents and uncles. When David was 7 years old, he had a domestic accident that resulted in a TBI classified as severe.

MRI performed after the injury showed multiple peripheral microhemorrhagic corticosubcortical foci in the right cerebral hemispheres, predominantly in the deep white matter of the right frontal lobe and bilateral posterior frontobasal regions, compatible with hemosiderin deposits associated with diffuse axonal injury. The last MRI, performed 2 months before the start of the present study, concluded that the participant’s injuries were stable: multiple foci of multifocal axonal injury of the supratentorial convexity and a small nodular hemorrhagic focus in the right middle cerebellar peduncle.

A neuropsychological assessment was carried out prior to the start of the study. Since the accident and up to that time the child had received 2 years of neuropsychological rehabilitation. At no time had there been any intervention aimed at social cognition or any school adaptation. In the Kaufman Brief Intelligence Test (K-BIT) (Kaufman, 1990) he obtained an IQ in the 95th percentile (88th percentile in vocabulary and 98th in matrices). In relation to attention, Trail Making Test-part A (Reitan, 1958; norms of Arango-Lasprilla et al., 2017) score placed the participant in the 15th percentile. In relation to executive functions, Trail Making Test-part B score was in the 14th percentile; the Five Digit Test (Sedó, 2007; norms of Rodríguez et al., 2012) in the 55th percentile; and the Tower of London Drexler (TOLDX) in the 10th percentile (Culbertson and Zillmer, 1998).

As for emotional recognition, the Ekman 60th Faces Test (Ekman and Friesen, 1976; norms of Gómez-Pérez et al., 2016) score was in the 70th percentile (anger 62th, disgust 97th, fear 10th, happiness 32th, sadness 65th and surprise 62th). Social knowledge were assessed with the Faux Pas Test (Baron-Cohen et al., 1999) and the score obtained placed the child in the 5th percentile. ToM and empathy was assessed by the Stories of Everyday Life test (Kaland et al., 2002; norms of Lera-Miguel et al., 2016) where the physical inference score was at the 29.8th and the mental inference at the 10th, and the Empathy/Systemizing Quotient (EQ-SQ) (Auyeung et al., 2009) where score was at the 12.9th percentile in EQ and 44th percentile in SQ.

During the interview with the parents and the child and the administration of the tests, problems of language comprehension or expression were discarded. His parents reported that, prior to TBI, David had excellent relationships with his siblings and classmates and would spend his free time playing sports and socializing with his friends. Now, David’s parents reported that he has deficits in most situations that involve (1) expressing positive emotions, (2) adapting to changes in plans and routine, and (3) initiate contact with schoolmates.

Concerning expressing emotions, the family detailed that David is always indifferent to any situation, even on those exceptional occasions when they consider him to be happy. Regarding the reaction to changes in plans, David’s parents stated he is too strict with his daily routines. If an activity is changed or a plan is not carried out, even if the family explains why, David does not understand and refuses to accept the situation. Finally, the family is concerned that David has no relationships with his classmates, he does not even greet them when he arrives at school. He attends school daily and participates in extracurricular activities but limits himself to doing what the teacher or the coach says without interacting with others. At recess, David prefers to be alone rather than with his classmates.

### 2.3. Context and approvals

The study was conducted face-to-face at the Mind, Brain, and Behaviour research center of the University of Granada. First, the principal researchers of the study met with the parents to explain the single-case study methodology and to specify the behaviors to be studied. Then, after determining the design and developing the specific intervention, a second meeting was held to explain all the details. Then, once the parents signed the “informed consent” document, the study began. During the first 2 weeks, a pilot study was conducted to familiarize the parents with the data collection process, after which the baseline data collection began. Finally, the interventions, which were conducted by the principal researchers, were carried out at the research center. The study was conducted following the Declaration of Helsinki and approved by the Human Research Ethics Committee of the University of Granada (N° certificate 706/CEIH/2018).

### 2.4. Interventions

The researchers designed and implemented three different interventions, one directed at each target behavior. Some activities were adapted from the “Reading a smile” program (Bornhofen and McDonald, 2010), while others were created to respond to the study objectives. The three interventions work on four types of content across 10 sessions, each lasting 40 min. Further details of some of these activities can be found in the Supplementary material. For further information, please contact the corresponding author.

Target Behavior 1: Expression of positive emotions

The aim was for David to express more positive emotions. To achieve this objective, the CS component that was worked on was the processing of emotions. The elements of the intervention were (a) differentiating between positive and negative emotions (Session 1), (b) identifying the emotions felt in hypothetical situations (Sessions 2–3), (c) learning how to express each emotion (Sessions 4–8), and (d) analyzing the expressions and behaviors shown from personal experiences with each of the emotions worked on and offering proposals for behavioral change (Sessions 9–10). Further details of these activities can be found in the Supplementary material. However, as an example we summarize here the essence of one of the activities carried out as part of this intervention:

- Activity title: Facial recognition.
- Objective: to learn to differentiate and express different emotions.
- Resources: images and the digital platform *mobbyt*.
- Method: the three areas of the face they should look at to recognize the emotion expressed by the person (forehead, eyes and mouth) are explained. Then, they are shown how these areas should look like for each of the emotions and they are asked to recognize the emotion that appears in the images. Finally, the child must express with his/her face the emotions that have been worked on.
- Emotions worked on: surprise, anger, sadness, fear, happiness, disgust, nervousness.

Target Behavior 2: Reacting to change in plans

The goal was for David to decrease his negative reactions and increase his positive reactions to changes that occur. To achieve this goal, two components of CS were trained: ToM and social knowledge. The intervention was mainly aimed at improving ToM so that David could understand others when they needed to make changes in plans or routines. In addition, training to improve social knowledge was also included at the end of the intervention. For this, he analyzed different reactions of himself and others to changes in plans and, when they were considered inappropriate, he had to propose alternatives. For the alternatives to be correct, the child had to take into account what others were thinking and feeling and that the response was appropriate for the particular social context. The contents of the program were (a) learning that other people may have different tastes or preferences from him (Sessions 1–3), (b) understanding that sometimes the plan must be altered for an unexpected reason (Sessions 4–5), (c) understanding that the plan can be altered because at that moment the others do not feel like it (Sessions 6–7), and (d) analyzing the behaviors shown in response to a change of plans and propose alternatives when such behaviors were considered inappropriate (Sessions 8–10). An example of the activities carried out in this intervention was:

- Activity title: Change the plan.
- Objective: To understand that the plan can be altered because others do not feel like it at that moment.
- Resources: stories, paper and pen.
- Method: read a story in which the characters had organized a plan but one of them does not feel like it and the plan is canceled. Answer several questions about how each character would feel.
- Example story:

Sofia is a 16-year-old girl. In her free time she loves going to the movies, it’s her favorite thing to do. Last week she was talking with Claudia, a friend from her neighborhood, and they agreed to go to the movies together next Saturday. When Saturday arrived, Claudia called Sofia and told her that she did not feel like going to the movies, and asked if they could make another plan. Finally, they went for a walk and an ice cream.

- Questions: How did Sofia feel when Claudia called her to change the plan? / How did Claudia feel?/How did they feel about the new plan?
- Last step: Think of a situation where a plan was changed because someone did not feel like it. How did it feel?

Target behavior 3: Greeting peers

In this case, the objective was for David to greet his classmates when they arrived at school. To achieve this goal, three components of CS were worked on: social knowledge, theory of mind and empathy. The contents were (a) learning the basic social rules of relating to others (greetings, farewells), asking how they are (Sessions 1–3), (b) analyzing the behavior of different characters through videos and texts, and deciding what could be done and what had to be changed (Sessions 4–7), (c) analyzing specific situations where he had not complied with the social rules and what he should have done (Sessions 8–9), and (d) analyzing how his behavior has changed since he started therapy and how he feels now (Session 10). An example of the activities carried out in this intervention was:

- Activity title: Is everything correct?
- Objective: Analyzing behavior of different characters, through videos and texts, and deciding what could be done and what had to be changed.
- Resources: video, paper and pen.
- Method: Watch a video or read a story in which a person engages in inappropriate behavior. Answer several questions about how the characters behaved. He then has to give an example of a situation in which he did not act as he should.
- If the video is very long, it is divided into 15-min periods and a section is worked on.
- Example of video: A Christmas Carol-Disney.

### 2.5. Measures

Measurements of the first and second behaviors were taken every day. Those of the third behavior were taken from Monday to Friday, because there is no school on weekends. In total, the study lasted 151 days and, if the weekends of the third behavior are not counted, the data were collected in 140 days.

Target behavior 1: the researchers gave the family a list of the positive emotions that David worked on in the intervention. The parents organized a daily meeting or assembly of the five family members to discuss the day’s best moment. Parents often discuss this topic with their children, so this meeting was common for the family. The parents asked their children what their best moment of the day was and to describe their emotion(s). To avoid the influence of siblings, David was always encouraged to be the first to answer. Both parents wrote down their children’s answers. Later, they reviewed and verified their notes to ensure they were identical. The dependent variable was the number of positive emotions expressed by David during each daily family meeting.

Target behavior 2: the parents wrote down David’s reactions and responses to altering a pre-established action plan or routine. To comply with the study design, the family drew up a weekly schedule specifying their activities. A few hours before the planned activity was due, the parents told David it would not take place, explaining the reason for the change. The dependent variables were (a) acceptance or rejection of the change and (b) the intensity of the negative reaction in the case of rejection. For measuring the first variable, David’s mother described his reaction as acceptance or rejection. For the second variable, the family was given a scale (0–10) to rate the intensity of the reaction. Specific examples of each level were detailed on the scale to facilitate a consistent rating. In addition, the researchers also reviewed the recordings to verify the score given to the behavior.

Target behavior 3: David’s mother recorded his behavior while waiting to enter the school with a pair of camera glasses (Banglin^©^ 1080P HD micro SD 32GB). Dependent variables were (a) the number of initiatives David took to greet his classmates and (b) how many of them he greeted before entering the classroom in the morning. For measuring both variables, the parents reviewed the recordings at home to record in a log: (a) whether David had the initiative to approach any classmate, and (b) how many children David greeted/interacted with. In addition, the researchers also reviewed the recordings to verify the score given to the behavior.

### 2.6. Data analysis

The Non-overlap of all pairs (NAP) statistic and split-half trend estimation method (S-HTEM) were applied to analyze quantitative variables. In addition, chi-square test (*X^2^*) results and a graphical representation of percentages in each phase were presented for categorical variables.

The NAP is a non-parametric technique to measure the non-overlap or ‘dominance’ of a quantitative variable between two phases. This analysis considers all possible overlaps between phases (baseline-intervention, intervention-withdrawal, baseline-withdrawal) as it provides a pairwise comparison between all first and second-phase data. Therefore, it could be interpreted as the percentage of non-overlapping data between the two phases (Manolov and Moeyaert, 2017). The NAP effect can be rated as weak (0–0.31), medium (0.32–0.84), or large (0.85–1) (Parker and Vannest, 2009; Parker et al., 2011). NAP was calculated using the online program http://singlecaseresearch.org/calculators/nap (Vannest et al., 2016). Graphical representation of the S-HTEM shows the baseline trend. A significant change is considered to have occurred when three or more consecutive measurement points deviate from the trend in the intervention or withdrawal phases (Lane and Gast, 2014; Manolov et al., 2016).

## 3. Results

Target Behavior 1: Expression of positive emotions

**Figure 1:**
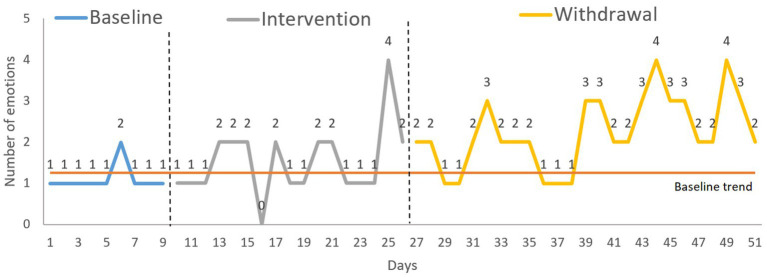
Target behavior 1: expression of positive emotions. Analysis: S-HTEM (split-half trend estimation method).

The NAP revealed a significant change in the number of positive emotions between phases B and A’ (medium effect) and A vs. A’ (large effect) (see Table 1). Inspection of the S-HTEM graph indicates that three consecutive assessment points were above the trendline in the intervention phase and 13 in the withdrawal phase (see Figure 1). Both analyses revealed a significant increase in the expression of positive emotions throughout the intervention, which maintained and strengthened the effect size in the withdrawal phase.

**Table 1:** Results of the non-overlap of all pairs (NAP) statistics for each quantitative dependent variable.

Target behavior order (quantitative variable)	Baseline-intervention (A vs. B)			Intervention-withdrawal (B vs. A´)			Baseline-withdrawal (A vs. A´)		
	NAP	CI90%	*p*	NAP	CI90%	*p*	NAP	CI90%	*p*
1st (Number of positive emotions expressed)	0.670	−0.063<>0.744	0.165	0.712	0.117<>0.733	**0.023**	0.864	0.354<>1	**0.001**
2nd (Intensity of negative reactions)	0.540	−0.251<>0.412	0.691	0.815	0.314<>0.947	**0.001**	0.836	0.306<>1	**0.003**
3rd (Number of classmates greeted)	0.883	0.351<>1	**0.002**	0.346	−0.676<>0.062	0.171	0.844	0.297<>1	**0.004**

NAP, non-overlap of all pairs; CI, Confidence interval.

Target Behavior 2: Reacting to change in plans

**Figure 2:**
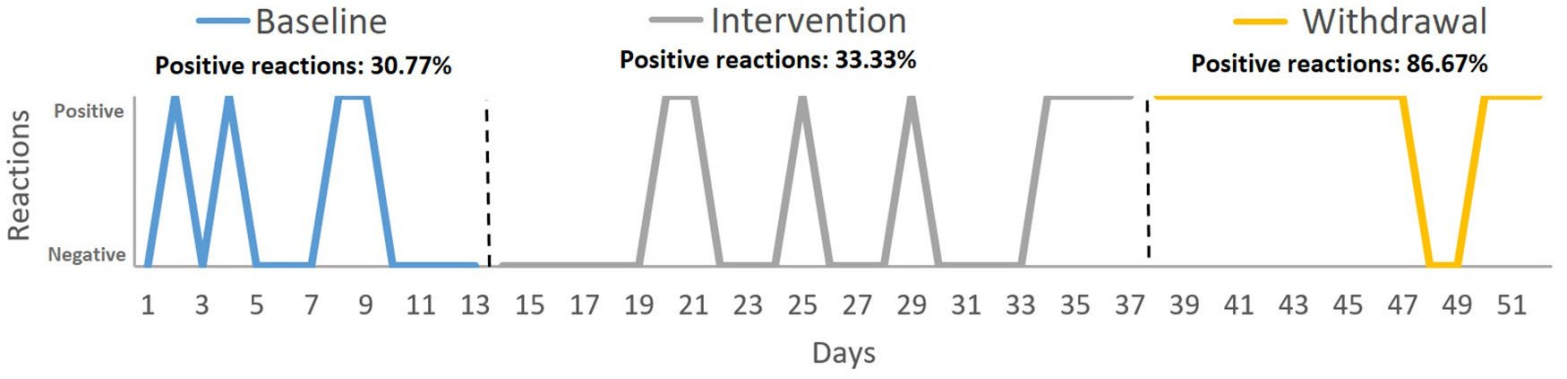
Target behavior 2: reacting to change in plans. Variable: acceptance or rejection of the change. Analysis: percentage of positive reactions with respect to the total number of reactions in each phase.

For the categorical variable *acceptance or rejection of changes*, the chi-squared test (*X^2^*) showed a significant change between phase B and A’ (*X*^2^ = 10.565, *p* = 0.001) and A vs. A’ (*X*^2^ = 9.123, *p* = 0.003). In addition, the graph showed that acceptance increased significantly in the withdrawal phase (86.67%) (see Figure 2).

**Figure 3:**
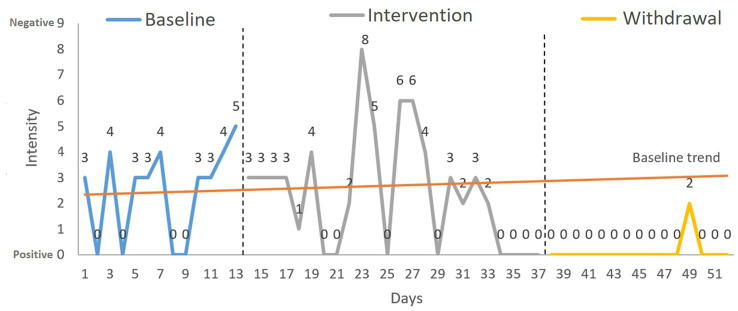
Target behavior 2: reacting to change in plans. Variable: intensity of the negative reaction. Analysis: S-HTEM (split-half trend estimation method).

Regarding the *intensity of negative reactions* when the change was rejected, the NAP showed a significant change between phase B and A’ and between A and A’ (see Table 1) with a medium effect size. The S-HTEM graph shows an increase in negative reactions in the intervention phase that declined at the end of this phase (the last four points indicated no negative reaction), a trend that continued throughout all but one of the days of the withdrawal phase (see Figure 3).

Target Behavior 3: Greeting peers

**Figure 4:**
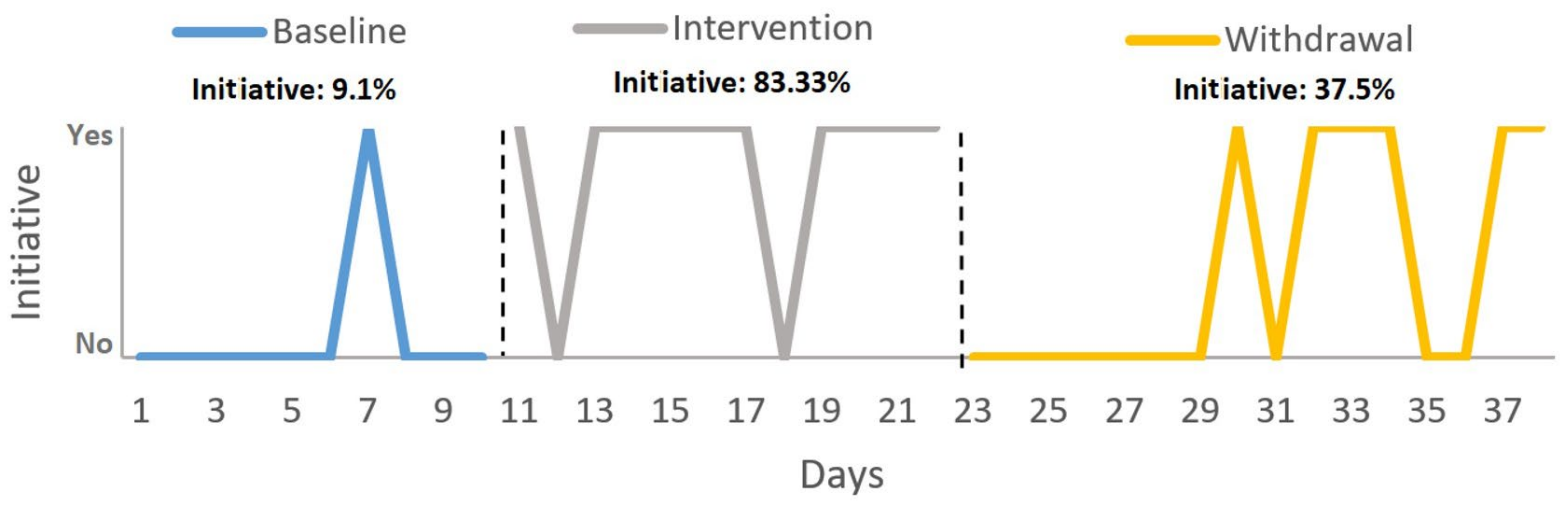
Target behavior 3: greeting peers. Variable: initiative to greet. Analysis: percentage of initiative with respect to the total number of initiative in each phase.

For the categorical variable *initiative to greet*, *X^2^* revealed significant changes between phases A and B (*X^2^* = 11.733, *p* = 0.001) and between phases B and A’ (*X^2^* = 5.882, *p* = 0.015). Figure 4 shows that the participant’s initiative to greet his classmates increased in the intervention phase, reaching 83.33%, but this decreased in the withdrawal phase (37.5%).

**Figure 5:**
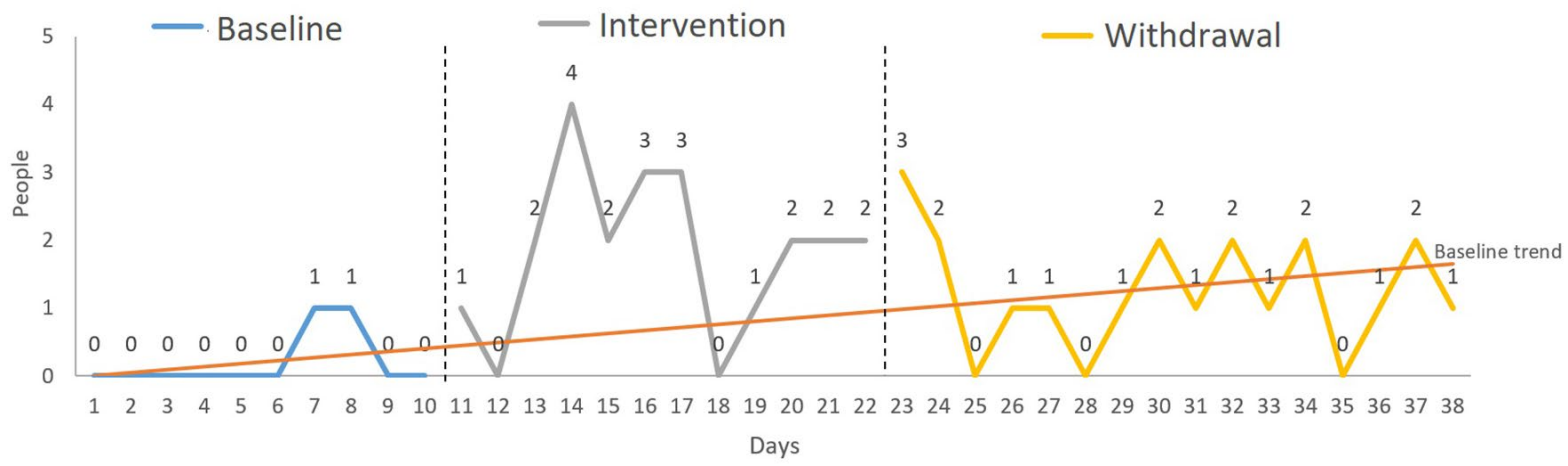
Target behavior 3: greeting peers. Variable: number of classmates greeted by David. Analysis: S-HTEM (split-half trend estimation method).

Regarding the *number of classmates that David greeted*, the NAP showed a significant (large effect size) increase between baseline and the other phases (A vs. B and A vs. A’) and no change between B and A’ (see Table 1). The S-HTEM graph shows more than three consecutive assessment points above the trendline in the intervention phase. Concerning the withdrawal phase, although many assessment points are above the line, these are not consecutive. This means that, although the treatment effect continued, it was not stable (see Figure 5).

## 4. Discussion

We aimed to determine the efficacy of three interventions to improve three behaviors — expression of positive emotions, reacting to changes in plans, and greeting peers — applied to a nine-year-old boy with TBI.

The first intervention was directed toward improving the expression of positive emotions. To achieve this objective, the CS component that was worked on was the processing of emotions. When assessed at baseline, David would only express one positive emotion. However, during and after the intervention, he managed to express three or four. This finding indicates that the treatment was impactful, producing a large effect (Parker and Vannest, 2009) that persisted after withdrawal. Many people, like David, find it difficult to recognize and express their and others’ emotions after TBI (Nahi et al., 2022), which can limit their social and personal development (Binder et al., 2019). For instance, Lancelot and Gilles (2019) showed that deficits in emotion recognition hinder the development of social knowledge, which is key for integration into everyday social life (Lancelot and Gilles, 2019). Moreover, most models suggest that emotion processing precedes and serves as a source of information for ToM (Mitchell and Phillips, 2015). Therefore, impairment in emotion processing can impede the correct development of ToM. Other authors also found that deficits in emotion recognition affect cognitive empathy (Blair, 2005). For this reason, the social participation of people with TBI is very limited or non-existent (Westerhof-Evers et al., 2019). Therefore, the scientific literature suggests that any social cognition rehabilitation program designed for people with TBI should be directed at treating problems with emotion recognition (Vallat-Azouvi et al., 2019). Furthermore, the literature points out that the person must recognize and express positive emotions before working with negative emotions, so initial rehabilitation should focus on these positive emotions (Mieles Toloza et al., 2020). However, most rehabilitation programs simultaneously work on positive and negative emotions (Rodríguez-Rajo et al., 2018). This study has shown the effectiveness of training designed to improve the recognition and expression of positive emotions, which alone was sufficient since the participant could already express negative emotions.

The second target was behavior was reacting to change in plans. The findings showed an increase in acceptance behaviors during the intervention and withdrawal phases. Furthermore, the intensity of the negative reaction shown when David did not accept change decreased in the intervention and withdrawal phases compared to baseline. This pattern of results reflects a clear improvement in his flexibility toward accepting change. On the few occasions when David expressed rejection toward changes, he did so more adaptively, with a less intense negative emotional response. In the intervention for this behavior, two components of SC were worked on: social knowledge and theory of mind. To our knowledge, no comparable published programs exist for people with TBI. However, compared with other interventions that have addressed various components of SC in other populations (e.g., people with schizophrenia), this program is considerably shorter. The intervention time is usually between 16 and 24 sessions of 1 h each (Penn et al., 2005; Nahum et al., 2014). Aside from the difference in specific characteristics of the population, the tailored and personalized activities could be key to its effectiveness. Considering that SC is not a unidimensional construct (Cassel et al., 2016), the training content and the participants’ deficits must be accurately defined (Domínguez Morales et al., 2002). Subsequently, rehabilitation should then be tailored according to the participants and their family’s characteristics and lifestyles (Turner-Stokes et al., 2005; Oberholzer and Müri, 2019).

Finally, the third intervention was aimed at the target behavior of greeting classmates. In the intervention for this behavior, three components of SC were worked on: social knowledge, theory of mind and empathy. The results showed that the intervention increased David’s tendency to greet and initiate interactions with his peers. However, this initiative decreased during the withdrawal phase. In terms of the number of people greeted by David, this increased rapidly from almost none at baseline to three or four during the intervention and then stabilized at just one or two on most days of the withdrawal phase. According to the NAP, this change means that the intervention had a medium effect by the end of the study. However, when considering the split-half trend estimation method, the graph indicates that the initial change was not sufficiently strong to be maintained during the withdrawal phase. Our findings show that David experienced two positive changes in his interactions with peers during the active training phase, taking more initiative to greet and to greet a greater number of his peers. Although the NAP found no significant difference between the intervention and withdrawal phases in terms of the number of greetings, visually, it can be seen that the effects did not show the desired level of stability. Once the intervention had finished, the exchange of greetings remained higher than in pre-treatment because a couple of peers continued to greet him even though David no longer showed the same initiative as in the previous phase. The trend described above may be due to the complexity of the targeted behavior. In particular, Cassel et al. (2016) review shows that the average duration of training programs that include several SC components is between 16 and 25 h. Moreover, social interaction is one of the biggest challenges faced by people with TBI (Snapp and Martin, 2020). Therefore, longer time frames are recommended for future interventions aimed at improving interactions with others due to the persistent nature of these debilitating social impairments resulting from pediatric TBI (Anderson et al., 2017; Lancaster et al., 2019).

## 5. Conclusion

Our results show the effectiveness of two interventions targeting the expression of positive emotions and reacting positively to change of plans in a nine-year-old boy with TBI. However, the intervention aimed at greeting peers was only effective during the training phase, so a longer protocol is recommended to achieve desired outcomes that are more persistent over time.

## 6. Limitations and strengths

This study has several limitations. First, we employed the A-B-A’ design, which, although effective in this population (Peyroux and Franck, 2019; Cassel et al., 2020), could be extended in future studies to include more phases (e.g., A-B-A-B). A more extensive study design will allow for obtaining a larger data set and increase internal validity. Second, while only the participant was blinded to the aims of the intervention, the parents and the researcher was aware of the phases of the study and inter-rater reliability was not conducted. In addition, the real-life observational measures that have been used are ecologically valid but have a high degree of subjectivity. Achieving potential improvements in this regard would be challenging but desirable for future studies. Third, concerning assessment and intervention, the use of machines is recommended. However, the feasibility of their use depends on the behaviors to be worked on and the participant’s characteristics. For this particular study, assessment and intervention using machines would be highly complex, considering the behaviors that have been targeted. Specifically, concerning the third behavior — improving interaction with others — any intervention must involve contact with others and not only work with a machine. Finally, it is recommended that future studies include replications (one original and three replications), specifying generalization measures for each phase.

A notable strength of this study is that it was conducted according to the standards of the SCED guidelines (Kratochwill et al., 2013; Tate et al., 2013), taking into account the Risk of Bias in Trials Scale N of 1 (RoBiNT) (2015) and the Single-Case Reporting Guidelines for Behavioural Interventions (SCRIBE) (2016). The RoBINT (2015) scale is composed of 15 items divided into two subscales: (1) Internal validity subscale (Items 1–7) and (2) External validity and interpretation subscale (Items 8–15). Concerning the assessment, each item is assigned a score between 0 and 2. The total scale score is 30 points, with 14 points allocated to the internal validity subscale and 16 points to the external validity and interpretation subscale. While most published single-case studies assessed with the RoBINT scale score less than 15 points (Beckers et al., 2020), this study scored 19 points and thus has a higher score than most published articles (see Supplementary Table 1).

Finally, the SCRIBE guide consists of 26 items covering all the factors to be considered when conducting a single case study. However, as with the RoBINT scale, studies that use the guide do not fully comply with all the items (MacIntosh et al., 2020), and even when it is stated that this guide has been taken into account in the study design, the considered items are not specified (Sauer-Zavala et al., 2019; Sniewski et al., 2022). In contrast, the present study complied with 24 of the items set out in the SCRIBE guidelines (see Supplementary Table 2).

## Data availability statement

The raw data supporting the conclusions of this article will be made available by the authors, without undue reservation.

## Ethics statement

The study was conducted in accordance with the Declaration of Helsinki, was approved by the Human Research Ethics Committee of the University of Granada (certificate no. 706/CEIH/2018) and parents signed the “informed consent.” Written informed consent to participate in this study was provided by the participants’ legal guardian/next of kin. Written informed consent was obtained from the individual(s), and minor(s)’ legal guardian/next of kin, for the publication of any potentially identifiable images or data included in this article.

## Author contributions

SR-G has been involved in all phases of the study and has carried out the intervention. NP contributed to the writing of the article. AnC collaborated in writing the data analysis and provided comments on drafts of the manuscript. AlC has led the study by participating substantially and directly in all phases of the research: design, intervention, data analysis and writing the article. All authors contributed to the article and approved the submitted version.

## Funding

This research was supported by the FPU16/03165 grant from the Spanish Ministry of Education, awarded to SR-G. The publication of this paper was supported by The University of Cadiz, Spain.

## Conflict of interest

The authors declare that the research was conducted in the absence of any commercial or financial relationships that could be construed as a potential conflict of interest.

## Publisher’s note

All claims expressed in this article are solely those of the authors and do not necessarily represent those of their affiliated organizations, or those of the publisher, the editors and the reviewers. Any product that may be evaluated in this article, or claim that may be made by its manufacturer, is not guaranteed or endorsed by the publisher.

## Abbreviations

SC: Social cognition
TBI: Traumatic brain injury
SCED: Single-case experimental designs
RoBiNT: Risk of Bias in Trials Scale N of 1
SCRIBE: Single-case reporting guideline in Behavioral Interventions.
NAP: Non-overlap of all pairs
S-HTEM: Split-half trend estimation method
