# Supplementary material for: Effectiveness of training in expressing positive emotions, reacting to change and greeting peers after childhood traumatic brain injury: a single-case experimental study
Source: Front Psychol. 2023 Jul 12;14:1195765. doi: 10.3389/fpsyg.2023.1195765 (PMC10369192; doi:10.3389/fpsyg.2023.1195765)
Supplement: Supplementary file 1 [file Table_1.pdf]

Supplementary Material

Improving interactions with others after traumatic brain injury: a single-case experimental study

Sandra Rivas-García[*], Nuria Paúl, Andrés Catena and Alfonso Caracuel

*** Correspondence:**

Sandra Rivas García. Faculty of Education Sciences, 11519 Puerto Real, Cádiz.

E-mail: srivasresearcher@gmail.com

1 Supplementary Figures and Tables

Table 1

Risk of Bias in N-of-1 Trials (RoBiNT) Scale Record Form (Tate et al. 2015)

<table>
<tr><th colspan="3">INTERNAL VALIDITY (IV) SUBSCALE</th><th colspan="3">SCORE</th></tr>
<tr><td rowspan="3">1</td><td rowspan="3">Design with control</td><td>2 Points: At minimum: ABAB with 4 phases; concurrent multiple-baseline design (MBD) with 6 phases, 3 tiers; alternating-treatments design (ATD) with 4 sets of alternating sequences; changing-criterion design (CCD) with 4 steps; for medical N-of-1: 3 x AB pairs</td><td rowspan="3">2</td><td rowspan="3">1</td><td rowspan="3">0</td></tr>
<tr><td>1 Point: ABA or 3 phase variant; concurrent MBD with 4-5 phases, 2 tiers; ATD with 3 sets of alternating sequences; CCD with 3 steps; for medical N-of-1: 2 x AB pairs</td></tr>
<tr><td>0 Points: AB; AB+follow-up; non-concurrent MBD; ATD with <3 sets of alternating sequences; CCD with <3 steps; nonwithdrawable treatment in ABA</td></tr>
<tr><td colspan="6">WHERE: Line 215 “An A-B-A’ fall-back/withdrawal design was applied”. See under the heading “Desing”</td></tr>
<tr><td rowspan="3">2</td><td rowspan="3">Randomisation</td><td>2 Points: Randomise: sequence (order) and/or onset (start point) for all phases (see manual for exceptions)</td><td rowspan="3">2</td><td rowspan="3">1</td><td rowspan="3">0</td></tr>
<tr><td>1 Point: Restricted randomisation (e.g., participants to blocks of sequences); counterbalancing</td></tr>
<tr><td>0 Points: No information; randomisation of other aspects of the study (e.g., stimulus materials)</td></tr>
<tr><td colspan="6">WHERE: Line 229-230 “We randomized the order of the three targeted behaviors and the starting day of the intervention phase for each”</td></tr>
<tr><td rowspan="3">3</td><td rowspan="3">Sampling of behaviour</td><td>2 Points: 5 or more data points in every phase with data presented</td><td rowspan="3">2</td><td rowspan="3">1</td><td rowspan="3">0</td></tr>
<tr><td>1 Point: at least 3 data points in every phase with data presented</td></tr>
<tr><td>0 Points: <3 data points in any phase</td></tr>
</table>

WHERE: Line 231 “a minimum of nine measures were required at baseline”. See under the heading “Desing”					
4	Blinding of people involved in the intervention	2 Points: Both participant and practitioner blind to phase of study. If technological intervention used, consult manual	2	**1**	0
		1 Point: Participant or practitioner blind to phase. If technological intervention used, consult manual			
		0 Points: Neither participant nor practitioner are blind to phase			
WHERE: Line 217-226 “The participant was blinded to the study design. At the baseline phase, the child was unaware that his behaviors were being recorded, one of which was captured by a video camera. This arrangement was used to avoid any intentional behavior modifications. At the beginning of the intervention phase, the family explained to the child that he would participate in a study aimed at people with TBI. However, neither the characteristics of the study nor the fact that certain behaviors would be assessed outside the session was explained to the child. Finally, the child did not know that his behaviors were being recorded in the withdrawal phase (as with the previous phases)”. See under the heading “Desing”					
5	Blinding of assessor(s)	2 Points: Assessors blind to all phases; use of computer/machine free from human involvement; outcomes self-report and participant is blind	2	1	**0**
		1 Point: Independent assessor(s), but not blind to phase			
		0 Points: Practitioner collects/extracts/scores/processes the data; no mention of blinding or independence of assessor(s)			
WHERE: Recording was carried out through (1) parental reports and (2) behavioural recordings. The researchers then analysed the records and recordings. Both the family and the researchers were aware of the study and the phases. The procedure is detailed in the Measures section of the article.					
6	Interrater agreement	2 Points: Machine-generated data or data sampled from ≥20% per condition, analysed and reported per condition, with ≥80% agreement (k≥0.6, etc)	**2**	1	0
		1 Point: A reasonably objective measure (as defined in the manual) used or agreement is ≥70% (k≥0.4) even if (a) data are not calculated and reported per condition and/or (b) <20% of data is sampled per condition			
		0 Points: Agreement <70% (k<0.4, etc); subjective measure used; consensus ratings alone; inter-rater agreement only reported for a previous study			
WHERE: Line 494-498 “The Non-overlap of all pairs (NAP) statistic and split-half trend estimation method (S-HTEM) were applied to analyze quantitative variables. In addition, chi-square test (X2) results and a graphical representation of percentages in each phase were presented for categorical variables”. See under the heading “Data analysis”					
7	Treatment adherence	2 Points: Machine-delivered intervention free from human implementation or adherence assessed (i) against a clear rating system, (ii) assessor is independent of practitioner/participant, (iii) ≥20% of is data sampled, (iv) resulting in ≥80% adherence	2	1	**0**
		1 Point: Adherence meets 2/4 criteria above, and includes (a) assessor independent of practitioner and (b) adherence ≥70%			
		0 Points: Adherence <70%; assessor not independent of			

		practitioner; components only loosely related to adherence			
WHERE: This study had three different interventions, one for each behaviour. Some activities have been adapted from the "Read a smile" program (Bornhofen and McDonald 2010) and others were created to respond to the objectives set. The three interventions work on four types of content in ten sessions. Each session lasts 40 minutes. The activities can be found in the supplementary material. For more information, please contact the corresponding author.					
**EXTERNAL VALIDITY AND INTERPRETATION (EVI) SUBCALE**			**SCORE**		
8	Baseline characteristics	2 Points: Analysis of baseline characteristics and age, sex, aetiology, severity of condition	2	**1**	0
		1 Point: Analysis of baseline characteristics or age, sex, aetiology, severity of condition			
		0 Points: No analysis of baseline conditions or incomplete listing of the four participant characteristics			
WHERE: Line 515-555. See under the heading "Results".					
9	Setting	2 Points: Description of general location and detailed description of the specific environment	**2**	1	0
		1 Point: Description of either general location or specific environment but details are sparse			
		0 Points: Neither general location nor specific environment are described			
WHERE: Line 108-133. See under the heading "Participant".					
10	Dependent variable (target behaviour)	2 Points: Target behaviour is operationally defined in precise terms and the method of measuring it is described	**2**	1	0
		1 Point: Target behaviour is operationally defined, but its description and/or method of measurement is not clear and precise			
		0 Points: Target behaviour is not operationally defined			
WHERE: Line 447-489. See under the heading "Measures".					
11	Independent variable (therapy/ intervention)	2 Points: Detailed description of content of the intervention including any equipment/manuals (for medical N-of-1: content of the agents, both active and placebo) and 3 procedural details: number, duration (dosage for medical N-of-1) and frequency of sessions	**2**	1	0
		1 Point: General description of content of intervention (and equipment/manuals) and 2/3 procedural details (number, duration/dosage, frequency)			
		0 Points: Intervention described in general terms; only identified as a treatment approach (e.g., "cognitive-behaviour therapy"); <2/3 procedural details			
WHERE: Line 336-443. See under the heading "Interventions".					
12	Raw data record	2 Points: Raw data record with a data point for every session/observation period. If ≥10 individual trials, complete raw data record for ≥3 cases	**2**	1	0
		1 Point: xIf ≥10 or more individual trials, complete raw data record for 2 cases, or provision of a data record but data aggregated/averaged across sessions/periods, or provision of data record but a priori decision not to record data for every session (e.g., multiple probe studies)			

		0 Points: No raw data reported; data only reported for selected phases, omitted data			
WHERE: Line 515-555. In this research, data collection for each of the interventions carried out has been daily. The data are reflected in the visual analysis. See under the heading “Results”.					
13	Data analysis	2 Points: Systematic visual analysis with specified protocol, or visual analysis aided by quasi-statistical techniques, or statistical analysis with rationale	**2**	1	0
		1 Point: Systematic/aided visual analysis with selection of analytic techniques, or statistical analysis but no rationale, or a priori decision re the level of the target behaviour constituting an empirically derived clinically meaningful change			
		0 Points: Visual inspection without data analysis; analysis not conducted on target behaviour; arbitrary selection of level of target behaviour			
WHERE: Line 492-514. Data analysis was based on several statistical techniques applicable to each of the target behaviours. Analyses were (1) non-overlap of all pairs (NAP), (2) split-half trend estimation method (S-HTEM), (3) Chi-square test (X2) and (4) percentage of behaviours in each phase in relation to the total number of recorded behaviours. See under the heading “Data analysis”					
14	Replication	2 Points: 1 original + 3 replications (direct inter-subject or systematic including settings, behaviours, practitioners, intervention)	2	1	**0**
		1 Point: 1 original + 1 or 2 replications (inter-subject or systematic)			
		0 Points: No replication			
WHERE: This study is not replicated.					
15	Generalisation	2 Points: Specified generalisation measure is probed in every phase	2	1	**0**
		1 Point: Specified generalisation measure is probed only in pre- and post-treatment phases			
		0 Points: No generalisation measures			
WHERE: The measure of generalisability is not specified in this study.					
**Internal Validity Subcale: 8/14**		**External Validity and Interpretation subcale: 11 /16**	**TOTAL SCORE: 19/30**		
