# Supplementary material for: Effectiveness of training in expressing positive emotions, reacting to change and greeting peers after childhood traumatic brain injury: a single-case experimental study
Source: Front Psychol. 2023 Jul 12;14:1195765. doi: 10.3389/fpsyg.2023.1195765 (PMC10369192; doi:10.3389/fpsyg.2023.1195765)
Supplement: Supplementary file 2 [file Table_2.pdf]

Supplementary Material

Improving interactions with others after traumatic brain injury: a single-case experimental study

Sandra Rivas-García*, Nuria Paúl, Andrés Catena and Alfonso Caracuel

*** Correspondence:**

Sandra Rivas García. Faculty of Education Sciences, 11519 Puerto Real, Cádiz.

E-mail: srivasresearcher@gmail.com

1 Supplementary Figures and Tables

TABLE 2

The Single-Case Reporting Guideline In BEhavioural Interventions (SCRIBE)

TOPIC		ITEM DESCRIPTION	WHERE
TITLE and ABSTRACT			
1	Title	Identify the research as a single-case experimental design in the title	Line 1. “Effectiveness of training in expressing positive emotions, reacting to change and greeting peers after childhood traumatic brain injury: a single-case experimental study”.
2	Abstract	Summarize the research question, population, design, methods including intervention/s (independent variable/s) and target behavior/s and any other outcome/s (dependent variable/s), results, and conclusions	Line 77-102. See under the heading “Abstract”
INTRODUCTION			
3	Scientific background	Describe the scientific background to identify issue/s under analysis, current scientific knowledge, and gaps in that knowledge base	Line 109-200. See under the heading “Introducction”
4	Aims	State the purpose/aims of the study, research question/s, and, if applicable, hypotheses	Line 193-200. See the last paragraph under the heading "Introduction".
METHOD			
DESIGN			
5	Design	Identify the design (e.g., withdrawal/reversal, multiple-baseline, alternating-treatments, changing-criterion, some combination thereof, or adaptive design) and describe the phases and phase sequence	Line 205-240. See under the heading “Design”

		(whether determined a priori or data-driven) and, if applicable, criteria for phase change	
6	Procedural changes	Describe any procedural changes that occurred during the course of the investigation after the start of the study	The procedure has not been modified in this study.
7	Replication	Describe any planned replication	This study has not been replicated
8	Randomization	State whether randomization was used, and if so, describe the randomization method and the elements of the study that were randomized	Line 104-106. Seethe last paragraph under the heading "Design".
9	Blinding	State whether blinding/masking was used, and if so, describe who was blinded/masked	Line 227-240. See under the heading “Design”
PARTICIPANT/S or UNIT/S			
10	Selection criteria	State the inclusion and exclusion criteria, if applicable, and the method of recruitment	Line 246-253. See under the heading “Selection criteria”
11	Participant characteristics	For each participant, describe the demographic characteristics and clinical (or other) features relevant to the research question, such that anonymity is ensured	Line 255-314. See under the heading “Participant characteristics”
CONTEXT			
12	Setting	Describe characteristics of the setting and location where the study was conducted	Line 317-333. See under the heading “Context and Approvals”
APPROVALS			
13	Ethics	State whether ethics approval was obtained and indicate if and how informed consent and/or assent were obtained	Line 318-333. See underthe heading “Context and Approvals”
MEASURES and MATERIALS			
14	Measures	Operationally define all target behaviors and outcome measures, describe reliability and validity, state how they were selected, and how and when they were measured	Line 447-489. See under the heading “Measures”
15	Equipment	Clearly describe any equipment and/or materials (e.g., technological aids, biofeedback, computer programs, intervention manuals or other material resources) used to measure target behavior/s and other outcome/s or deliver the interventions	Line 447-489. See under the heading “Measures”
INTERVENTIONS			
16	Intervention	Describe the intervention and control condition ineach phase, including how and when they were actually administered, with as much detail as possible to facilitate attempts at replication	Line 336-443. See under the heading “Intervention”

17	Procedural fidelity	Describe how procedural fidelity was evaluated in each phase	Fidelity was not calculated
ANALYSIS			
18	Analyses	Describe and justify all methods used to analyze data	Line 492-514. See under the heading "Data analysis"
RESULTS			
19	Sequence completed	For each participant, report the sequence actually completed, including the number of trials for each session for each case. For participant/s who did not complete, state when they stopped and the reasons	Line 515-556. See under the heading "Results"
20	Outcomes and estimation	For each participant, report results, including raw data, for each target behavior and other outcome/s	Line 515-556. See under the heading "Results"
21	Adverse events	State whether or not any adverse events occurred for any participant and the phase in which they occurred	Line 515-556. See under the heading "Results"
DISCUSSION			
22	Interpretation	Summarize findings and interpret the results in the context of current evidence	Line 558-779. See under the heading "Discussion"
23	Limitations	Discuss limitations, addressing sources of potential bias and imprecision	Line 793-862.
24	Applicability	Discuss applicability and implications of the study findings	Line 793-901. See under heading "Limitations and Strengths"
DOCUMENTATION			
25	Protocol	If available, state where a study protocol can be accessed	See under the heading "Supplementary material"
26	Funding	Identify source/s of funding and other support; describe the role of funders	See under the heading "Funding"
