# Supplementary material for: Effectiveness of training in expressing positive emotions, reacting to change and greeting peers after childhood traumatic brain injury: a single-case experimental study
Source: Front Psychol. 2023 Jul 12;14:1195765. doi: 10.3389/fpsyg.2023.1195765 (PMC10369192; doi:10.3389/fpsyg.2023.1195765)
Supplement: Supplementary file 3 [file Table_3.pdf]

Supplementary Material

Improving interactions with others after traumatic brain injury: a single-case experimental study

Sandra Rivas-García*, Nuria Paúl, Andrés Catena and Alfonso Caracuel

*** Correspondence:**

Sandra Rivas García. Faculty of Education Sciences, 11519 Puerto Real, Cádiz.

E-mail: srivasresearcher@gmail.com

1 Supplementary Data

In this study, the researchers created three interventions, each targeting one target behaviour: (1) Expression of positive emotions, (2) Behavioural flexibility and (3) Interaction with classmates. The three interventions consist of four blocks of content and have a duration of 10 sessions of 40 minutes each. The applications used for the development of the activities were Mobbyt, Kahoot, Quizizz and Youtube.

**Target Behavior 1: Expression of positive emotions**	
The aim was for David to express more positive emotions	
**Contents**	**Activity**
Differentiating between positive and negative emotions	1. Emotionary<br>2. What emotion have we felt?<br>3. Sorting emotions
Identifying the emotion felt in hypothetical situations	4. What emotion do we feel in each situation?<br>5. Can we say everything we think?
Learning how to express each emotion	6. Facial recognition<br>7. What emotion does each character feel?
Analysing the expression and behaviour in personal experiences with each of the emotions worked on and proposing proposals for behavioural change	8. Analysing personal experiences

**Target Behavior 2: Reacting to changes in plans**
The aim was for David to decrease his negative reactions and increase his positive reactions to the changes that had occurred (behavioural flexibility)

**Contents**	**Activity**
Learning that other people, may have different tastes or preferences than he does	1. Diverse tastes<br>2. List of tastes<br>3. Different tastes
Understanding that sometimes the plan must be altered for an unexpected reason	4. What has happened?<br>5. When did something similar happen?
Understanding that the plan can be altered because at that moment the others do not feel like it	6. What does the character feel?<br>7. Changing the plan.
Analysing the behaviour that has taken place in situations where the behaviour has been modified and proposing how it should be	8. Analysing personal experiences.

**Target Behavior 3: Greeting peers**	
The aim was for David to improve personal interaction with classmates	
**Contents**	**Activity**
Learning the basic social rules of relating to others (greetings, farewells, asking how they are)	1. Rules social.<br>2. Steps to take before saying or doing something
Analysing the behaviour of different characters, through videos and texts, and deciding what could be done and what had to be changed	3. Is everything correct?<br>4. What to do when I feel negative emotions?
Analysing specific situations where he had not complied with the social rules and what he should have done	5. Analysing personal experiences.
Analysing how his behaviour has changed since he started therapy and how he feels now	6. How have we moved forward?

1.1 Examples of some activities

Activity 1. Emotionary

Method: show each emotion, explain what it means and given an example. Afterwards, the participant is asked to match emotion with its definition.

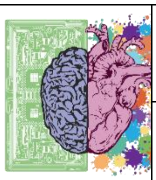	**Initial Level**	**Advanced Level**
	Link: https://mobbyt.com/videojuego/educativo/?Id=231857	Link: https://mobbyt.com/videojuego/educativo/?Id=177427

Activity 2. What emotion did we feel?
Method: the participant is asked to given an example of each of the emotions worked on.
Materials: Emotions worked out:

SURPRISE	SADNESS	HAPPINESS	ANNOYANCE
ANGER	FEAR	DISGUST	DISCOMFORT
NERVOUSNESS	HOPE	CONTENTMENT	DESPONDENCY
PRIDE	SHAME	LOVE	WORRY
LOVESTRUCK	HEARTACHE	HATRED	PHOBIC
EUPHORIA	JEALOUSY	BOREDOM	FRIENDLINESS
DESPAIR	ENVY	REJECTION	NOSTALGIA
DISAPPOINTMENT	RAGE	ENTHUSIASM	ANGUISH

Activity 3. Sorting emotions.
Method: This activity is divided into two parts. In the first part, the participant has to classify the emotions worked on in three categories: (1) Positive, (2) Negative and (3) Both, in this category we include emotions that can be confusing. In addition, several examples are specified in order to classify them. In the second part, the participant adds emotions.

**POSITIVE**	**NEGATIVES**	**BOTH**

Activity 4. What emotion is felt in each situation?

Method: a situation with four emotions is shown. The participant has to choose the emotion that can be felt in each situation.

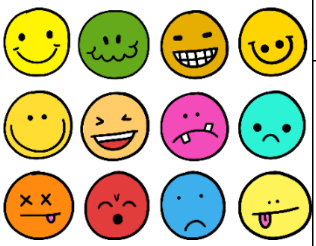	**Initial Level**	**Advanced Level**
	Link: https://create.kahoot.it/share/que-emocion-se-siente-en-cada-situacion/3c734e1a-ee78-4aa6-93b8-316b3dfe74f3	Link: https://create.kahoot.it/share/emocionario-que-emocion-se-siente-en-cada-situacion/d9f656eb-7a44-4275-ae11-2ee147e4b1ba

Activity 5. Can we say everything we think?

Method: various situations are shown where the participant has to identify the emotion that the person will feel and determine whether that comment is appropriate or not.

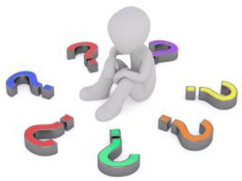	Link: https://quizizz.com/join/quiz/62ebed1c65f056001ef341cc/start?studentShare=true	

Activity 6. Facial recognition.

Method: the three areas of the face they should look at to recognize the emotion expressed by the person (forehead, eyes and mouth) are explained. Then, they are shown how these areas should look like for each of the emotions and they are asked to recognize the emotion that appears in the images. Finally, the child must express with his/her face the emotions that have been worked on.

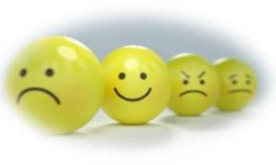	Link: https://mobbyt.com/videojuego/educativo/?Id=177437	

Activity 7. What emotion does each character feel?

Method: several videos are shown in which they have to recognize the emotion expressed by each character.

Materials: the stories used were: (1) Mickey's Christmas Carol, (2) Rock, Paper, Scissors, (3) Empathy, Hedgehog Story and (4) For Sale Mouse.
